# Program in BASIC to combine the data from two channels of an integrator, and its use in the calculation of residues per 1000 residues amino-acid analyses

**DOI:** 10.1155/S146392468200008X

**Published:** 1982

**Authors:** K. John Cronin, Paul G. Clarke, John J. Harding

**Affiliations:** 1 Nuffield Laboratory of Ophthalmology University of Oxford Walton Street Oxford OX2 6AW UK; 2 Trivector Systems Ltd Sunderland Road Sandy Bedfordshire SG19 1RB UK


Journal of Automatic Chemistry, Volume 4, Number 1 (January–March 1982), pages 25–31

### Program in BASIC to combine the data from two channels of an integrator, and its use in the calculation of residues per 1000 residues for amino-acid analyses

**K. John Cronin**

*Nuffield Laboratory of Ophthalmology, University of Oxford, Walton Street, Oxford OX2 6AW, UK,*

**Paul G. Clarke**

*Trivector Systems Ltd, Sunderland Road, Sandy, Bedfordshire SG19 1RB, UK,*

**and John J. Harding**

*Nuffield Laboratory of Ophthalmology, University of Oxford, Walton Street, Oxford OX2 6AW, UK*

### Introduction

Single-channel integrators have been used with gas-liquid chromatography (g.l.c.) equipment and amino-acid analysers for some years and are adequate for many g.l.c. applications. During amino-acid analysis with the conventional ninhydrin system most amino-acids give a blue product but others, notably proline and hydroxyproline, give a yellow product. These products are measured separately, so for manual calculation a two-pen, or three-pen, recorder has always been provided. Attempts to use a single-channel integrator for amino-acid analysis are inevitably unsuccessful. Measuring just the blue channel at 570 nm leads to gross inaccuracies for the small peaks of proline and hydroxyproline; combining the two colorimeter outputs leads to base-line problems, as does the use of an intermediate wavelength.

There are now integrators available that enable the output from two or more channels to be integrated, but it is not always possible to combine the results from two channels. Amino-acid analysis results for proteins have been presented as residues per 1000 residues (or per 100 residues)—this method was first introduced as an aid to comparative studies and for this it is necessary to take data for proline, and hydroxyproline if present, from the 440 nm channel and combine it with data for all the other amino-acids from the 570 nm channel.

This paper presents a program that allows the stored data from two channels of a Trilab 3 integrator to be combined to give a full amino-acid analysis expressed in residues/1000 residues. The operator may elect to omit earlier blocks of data to reach an appropriate standard, and may also reassign peaks that were wrongly assigned in the automatic mode.

With minimal modification the program could be adapted to combine data from two g.l.c. runs on a single sample and express results in any convenient form.

### Materials and methods

The outputs from the 570 nm and 440 nm colorimeters of an amino-acid analyser (the LKB 4400, produced by LKB Ltd, Milton Road, Cambridge, UK) are taken via head amplifiers into two of the four channels of a Trilab 3 integrator (Trivector Systems Ltd, Sunderland Road, Sandy, Bedfordshire, UK).

*All correspondence should be sent to John J. Harding at the Nuffield Laboratory of Ophthalmology.*

The manufacturer's operating system and chromatography programs are used for data collection, immediate print-out and storage on mini-cassettes (Philips Data Systems Ltd).

After data collection the manufacturer's basic compiler (Version 5.9) was loaded, followed by the program listed in figure 1.

### The program

The REM statements make the program almost self-explanatory (figure 1). In line 40 the string 'B' represents the three letter codes for the expected amino-acids in order of elution; individual codes can then be extracted using the substring function (for example, line 120). Line 45 sets up the arrays for the two channels with the first array also serving for the assembly of the print-out. Lines 50 to 90 feed in the amount of each amino-acid in the standard analysis: 5 nmol for all amino-acids except hydroxyproline (10 nmol). The program moves on to line 200 and the operator is asked to load the DATA tape, and then the tape is opened (subroutine 11000 to 11020). The operator next has the opportunity to bypass any early runs (lines 231–235 and subroutine 17000 in figure 1; see the print-out in figure 2). The operator is then asked to enter the total number of runs before the standard data is read from the tape (subroutine 12000). Subroutine 11130 is also used at this stage—it converts strings that have been stored as numbers back to strings. The computer checks the channel before printing-out the number of the sample used as a standard. Lines 264 and 266 transfer the data for hydroxyproline and proline from the B channel array into the appropriate positions in the A channel. Lines 267–275, with subroutine 2000, allow new areas to be fed in for selected peaks where the automatic assignment has been incorrect.

Faulty assignment is usually caused by drifting retention times while the analyser is running overnight. Lines 278 to 330 then provide calculation of response factors for the standard and subroutine 100 allows them to be printed-out if required. From line 340 the program allows calculation of each run using subroutines 14000, 18200 and 11130 to read the data, the header and to change stored numbers to strings (line 14035 converts the retention-time units from seconds to minutes). Both channels are accessed before going to subroutine 17000 which bypasses the data for unnamed peaks. The areas for hydroxyproline and

0142–0453/82/0401 0025 $05·00 © 1982 Taylor & Francis Ltd proline, peaks 1 and 2 on the B channel, are transferred into the main channel array to be the first and seventh peaks respectively (lines 364 and 365). Next comes the reassignment routine (lines 367 to 376 including subroutine 2000), which allows new areas to be assigned to any of the amino-acids and sets the retention times of reassigned peaks to zero as a reminder. Then the concentrations are calculated (lines 379–400), and converted to residues/1000 residues (lines 430 to 730) before the report is printed (subroutine 16000) the array cleared (subroutine 18000) and the tape closed (line 999).

### The print-out

A print-out demonstrating the options available is shown in figure 2. After the invitation to load a DATA tape the operator was asked if he wished to skip some blocks. He chose to do so as the first two chromatographic separations were poor. The print-out gave the sample numbers for the bypassed data and then after being told the total number of runs proceeded with the calibration. The operator chose to reassign three peaks in the basic region (HYL, HIS and LYS) which had been incorrectly assigned; and chose to have the standard concentrations, response factors and peak numbers printed. Moving on to the analysis of a lens protein hydrolysate again, the operator was invited to reassign peaks and did so choosing to set hydroxyproline to zero and to reassign areas in the basic region (figure 3), taking the areas from the automatic print-out table of unassigned peaks. The results were then printed as shown in figure 3 giving retention times (zero if peak either absent or reassigned), names, areas, concentrations (nmol) and residues/1000 residues.

### Discussion

Changes to line 700 would enable the results to be presented in a different form, for example residues/100 residues and with a few extra lines residues/peptide or residues/g could be calculated. Exchange of the routines used to read data from tape would permit the use of this program with other microcomputer systems programmable in BASIC that are used as integrators. This program should have wide applicability to systems where data from two channels must be merged into a single analysis, whether the two channels represent two features of a single run, or two g.l.c., high-performance liquid chromatography or other chromatographic separations.

### Acknowledgements

The authors are grateful to Mr K. C. Rixon for skilled technical assistance with the amino-acid analyser.

```

10 REM AMINO ACID ANALYSIS RES/1000 PROGRAMME

20 REM PGC/JJH MAR/APR 1981

30 ?"Amino acid analysis res/1000 3.0 *kjc/pgc/jjh 1981"

35 G=0

40 B$="HYPASPTHRSERHSEGLUPROGLYALACYSVALMETILELEUTYRPHEHYLHISLYSARG"

45 DIM A1(23,6),B1(5,6)

46 DIM H3(25)

50 REM SET UP STANDARDS FOR CHAN A AND B

55 A2=20

60 B2=2

65 FOR I=1 TO A2

70 A1(I,2)=5

75 NEXT I

80 A1(1,2)=10

85 A1(10,2)=5

90 B1(1,2)=10

95 B1(2,2)=5

99 GOTO 200

100 C$=" = "

101 INPUT ("Do you wish to see STD concentrations (Y/N) ?")X$

102 IF X$<>"Y" THEN 195

105 ?"Channel A: "

110 FOR I=2 TO A2

115 J=I*3-2

120 A$= SUBSTR$(B$,J,3)

130 ?A$ & C$;A1(I,2),A1(I,0)[6.3],I

140 NEXT I

150 ?

160 ? "Channel B: "

170 ?"HYP" & C$;B1(1,2),A1(1,0)[6.3],"1"

180 ?"PRO" & C$;B1(2,2),A1(7,0)[6.3],"2"

190 ?

195 RETURN

```

*Continued on pages 27–29*

*Figure 1. BASIC program combining data from two channels and calculating amino-acid analysis results as residues/1000 residues.*

```

200 REM MASTER SEGMENT

210 ?"Please load DATA tape and press (RETURN)";

220 INPUT X$

230 GOSUB 11000:REM OPEN TAPE

231 INPUT("DO YOU WANT TO SKIP THE FIRST n BLOCKS? (YES/NO) ")Y$

232 IF Y$="NO" GOTO 240

233 INPUT("ENTER No. OF BLOCKS TO BE BYPASSED ")N

234 FOR K= 1 TO N

235   GOSUB 17000

236 NEXT K

240 INPUT("How many pairs of runs including STD? ")R

250 GOSUB 12000:REM READ STD A(B)

260 GOSUB 12000:REM READ STD B(A)

264 A1(1,1)=B1(1,1)

266 A1(7,1)=B1(2,1)

267 REM STD PEAK REASSIGNMENT

269 INPUT("Do you want to reassign STD peaks or areas? (Y/N) ")Z$

270 IF Z$="N" GOTO 278

272 GOSUB 2000:REM REASSIGN

273 A1(E,1)=A

275 GOTO 269

278 REM CALCULATE RESPONSE FACTORS

280 FOR I=1 TO A2

290 A1(I,0)=A1(I,1)/A1(I,2)

300 NEXT I

325 REM RESPONSE FACTOR= AREA/CONC.

330 GOSUB 100

340 REM CALCULATE EACH RUN IN TURN

345 FOR K=1 TO R-1

350 GOSUB 14000:REM READ SAMPLE A (B)

360 GOSUB 14000:REM READ SAMPLE B (A)

363 FOR J=1 TO 3

364 A1(1,J)=B1(1,J)

365 A1(7,J)=B1(2,J)

366 NEXT J

367 REM SAMPLE PEAK ASSIGNMENT

369 INPUT("Do you want to reassign SAMPLE peaks or areas? (Y/N) ")Z$

370 IF Z$="N" GOTO 379

372 GOSUB 2000:REM REASSIGN

374 A1(E,3)=A

375 A1(E,2)=0.0

376 GOTO 369

379 REM CALCULATE CONCNS

380 FOR I=1 TO A2

386 IF A1(I,0)=0 THEN 400

390 A1(I,4)=A1(I,3)/A1(I,0)

400 NEXT I

430 REM CONC= AREA/RESPONSE FACTOR

650 F=0

660 REM CALCULATE TOTAL CONCN

670 FOR I= 1 TO A2

680 F=F+A1(I,4)

690 NEXT I

695 IF F=0 THEN F=1000

700 REM CALCULATE CONC/TOTAL*1000 FOR EACH PEAK

705 T=0

710 FOR I=1 TO A2

720 A1(I,5)=A1(I,4)*1000/F

725 T=T+A1(I,5)

```

*Continued on pages 28 and 29*

```

730 NEXT I

740 REM PRINT REPORT

750 GOSUB 16000

755 ?

756 ? "TOTAL RESIDUES= ";T[6.3]

757 ?

758 GOSUB 18000:REM CLEAR

760 NEXT K

999 CLOSE #DATA

1000 END

2000 REM REASSIGNMENT

2020 INPUT("Type NUMBER of peak to be reassigned ")E

2024 P=3*E-2

2025 ?SUBSTR$(B$,P,3);

2030 INPUT("   Type its AREA ")A

2040 RETURN

11000 REM OPEN TAPE

11010 OPEN IN#,DATA,TAPE,RAN,"GLC RESULTS"

11020 RETURN

11130 H1(1)=H1(1)+64

11132 H1(0)=1

11134 CHANGE H1 TO H1$

11138 H2(0)=3

11140 CHANGE H2 TO H2$

11150 H3(0)=25

11160 CHANGE H3 TO H3$

11170 H4(0)=2

11180 CHANGE H4 TO H4$

11190 H5(0)=1

11200 CHANGE H5 TO H5$

11210 H6(0)=5

11220 CHANGE H6 TO H6$

11230 H7(0)=10

11240 CHANGE H7 TO H7$

11250 RETURN

12000 REM READ STD FROM A & B

12005 GOSUB 18200:REM READ HEADER

12008 IF G=1 THEN 999

12010 GOSUB 11130:REM CHANGE NO TO $

12020 IF H1$="B" THEN 13030

12030 ?"CALIBRATION OF CHAN A WITH "& H2$ & H3$

12050 FOR I= 1 TO H8

12060 INPUT #DATA,N1,N2,N3,A1(I,1),N5

12070 NEXT I

12075 GOTO 13075

13030 ?"CALIBRATION OF CHAN B WITH " &H2$ & H3$

13050 FOR I= 1 TOH8

13060   INPUT #DATA,N1,N2,N3,B1(I,1),N5

13070 NEXT I

13075 GOSUB 17000:REM SKIP

13080 RETURN

14000 REM READ SAMPLE A & B

14005 GOSUB 18200:REM READ HEADER

14008 IF G=1 THEN 999

14010 GOSUB 11130:REM CHANGE NO. TO $

14012 IF H1$="B" THEN 15015

14015 S1$=H2$

14020 FOR I= 1 TO H8

14030   INPUT #DATA,A1(I,1),N2,A1(I,2),A1(I,3),N5

14035 A1(I,2)=A1(I,2)/60

```

*Continued on page 29*

```

14040 NEXT I

14045 GOTO 15045

15015 S2$=H2$

15020 FOR I= 1 TO H8

15030   INPUT#DATA,B1(I,1),N2,B1(I,2),B1(I,3),N5

15035   B1(I,2)=B1(I,2)/60

15040 NEXT I

15045 GOSUB 17000:REM SKIP

15050 RETURN

16000 REM PRINT REPORT

16020 ? "SAMPLES "&S1$ &"   "&S2$

16030 ?"      RET TIME    NAME     AREA     CONCN     RESIDUES/1000"

16040 FOR I=1 TOA2

16050 J=3*I-2

16060 A$=SUBSTR$(B$,J,3)

16070 ?A1(I,2)[6.3],

16080 ?A$,

16090 ?A1(I,3)[6.3],A1(I,4)[6.3],A1(I,5)[6.3]

16100 NEXT I

16110 RETURN

17000 REM IGNORE NEXT SAMPLE ON TAPE

17010 GOSUB 18200:REM READ HEADER

17011 IF G=1 THEN 999

17014 GOSUB 11130:REM CHANGE NO. TO $

17016 IF H5$<>"E" THEN ?"Skipping "&H2$&H3$

17020 FOR I=1 TO H8

17030   INPUT# DATA,N1,N2,N3,N4,N5

17040 NEXT I

17050 RETURN

18000 REM CLEAR TABLE

18010 FOR I=1 TO 23

18020 FOR J=1 TO 5

18030 A1(I,J)=0

18040 NEXT J

18050 NEXT I

18060 FOR I=1 TO 5

18070 FOR J=1 TO 5

18080 B1(I,J)=0

18090 NEXT J

18100 NEXT I

18110 RETURN

18200 INPUT #DATA,H1(1)

18210 IF H1(1)>=1 THEN IF H1(1)<=4 THEN 18240

18220 G=1

18230 RETURN

18240 INPUT #DATA,H2(1),H2(2),H2(3)

18250 FOR I=1 TO 25

18260 INPUT #DATA,H3(I)

18270 NEXT I

18280 INPUT #DATA,H4(1),H4(2)

18290 INPUT #DATA,H5(1)

18300 INPUT #DATA,H6(1),H6(2),H6(3),H6(4),H6(5)

18310 FOR I=1 TO 10

18320 INPUT #DATA,H7(I)

18330 NEXT I

18340 INPUT #DATA,H8

18350 RETURN

```

*End of figure 1.*

```

Amino acid analysis res/1ØØØ 3.Ø *kjc/pgc/jjh 1981

Please load DATA tape and press (RETURN)?

DO YOU WANT TO SKIP THE FIRST n BLOCKS? (YES/NO) YES

ENTER No. OF BLOCKS TO BE BYPASSED 8

Skipping BØ7

Skipping AØ7

Skipping BØ8

Skipping AØ8

How many pairs of runs including STD? 7

CALIBRATION OF CHAN B WITH BØ9

CALIBRATION OF CHAN A WITH AØ9

Do you want to reassign STD peaks or areas? (Y/N) Y

Type NUMBER of peak to be reassigned 17

HYL   Type its AREA 682

Do you want to reassign STD peaks or areas? (Y/N) Y

Type NUMBER of peak to be reassigned 18

HIS   Type its AREA 7Ø3

Do you want to reassign STD peaks or areas? (Y/N) Y

Type NUMBER of peak to be reassigned 19

LYS   Type its AREA 775

Do you want to reassign STD peaks or areas? (Y/N) N

Do you wish to see STD concentrations (Y/N) ?Y

Channel A:

ASP =  5             1Ø1.279       2

THR =  5             122.148       3

SER =  5             129.322       4

HSE =  5             1Ø5.412       5

GLU =  5             127.212       6

PRO =  5              56.243       7

GLY =  5             148.625       8

ALA =  5             145.987       9

CYS =  5              69.5Ø1       1Ø

VAL =  5             135.159       11

MET =  5             15Ø.328       12

ILE =  5             151.591       13

LEU =  5             162.134       14

TYR =  5             139.343       15

PHE =  5             152.325       16

HYL =  5             136.4ØØ       17

HIS =  5             14Ø.6ØØ       18

LYS =  5             155.ØØØ       19

ARG =  5             123.Ø37       2Ø

Channel B:

HYP =  1Ø             28.Ø66      1

PRO =  5              56.243      2

```

*Figure 2. Print-out for the standard.*

```

Do you want to reassign SAMPLE peaks or areas? (Y/N) Y

Type NUMBER of peak to be reassigned 1

HYP   Type its AREA 0

Do you want to reassign SAMPLE peaks or areas? (Y/N) Y

Type NUMBER of peak to be reassigned 18

HIS   Type its AREA 3075

Do you want to reassign SAMPLE peaks or areas? (Y/N) Y

Type NUMBER of peak to be reassigned 19

LYS   Type its AREA 5078

Do you want to reassign SAMPLE peaks or areas? (Y/N) N

SAMPLES A11   B11

```

| RET TIME | NAME | AREA | CONCN | RESIDUES/1000 |
|---|---|---|---|---|
| .000 | HYP | .000 | .000 | .000 |
| 14.450 | ASP | 8020.239 | 79.189 | 115.108 |
| 16.550 | THR | 3516.942 | 28.793 | 41.852 |
| 17.650 | SER | 7243.011 | 56.008 | 81.412 |
| .000 | HSE | .000 | .000 | .000 |
| 21.150 | GLU | 11767.428 | 92.503 | 134.461 |
| 23.150 | PRO | 1940.265 | 34.498 | 50.145 |
| 26.800 | GLY | 7315.697 | 49.223 | 71.549 |
| 28.100 | ALA | 4479.188 | 30.682 | 44.599 |
| .000 | CYS | .000 | .000 | .000 |
| 33.200 | VAL | 4927.030 | 36.453 | 52.988 |
| 36.800 | MET | 2201.389 | 14.644 | 21.286 |
| 40.000 | ILE | 4502.904 | 29.704 | 43.178 |
| 41.450 | LEU | 7914.270 | 48.813 | 70.954 |
| 46.500 | TYR | 4979.409 | 35.735 | 51.944 |
| 48.250 | PHE | 5970.130 | 39.193 | 56.971 |
| .000 | HYL | .000 | .000 | .000 |
| .000 | HIS | 3075.000 | 21.871 | 31.791 |
| .000 | LYS | 5078.000 | 32.761 | 47.621 |
| 75.400 | ARG | 7122.040 | 57.885 | 84.141 |

TOTAL RESIDUES=   1000.000

*Figure 3. Print-out for lens protein.*

